# Albuminuria

**Published:** 1890-02-01

**Authors:** 


### The Practitioner's Outlook and Retrospect.

[*The Editor will be glad to receive offers of co-operation and contributions from members of the profession. There is no doubt that much valuable material full of interest and instruction is at present lost to science. This is largely so in the case of* (1) *the younger and less-known members of the hospital staff,* (2) *those connected with cottage hospitals, and* (3) *the great body of medical practitioners not attached to any institution. The co-operation of all is cordially invited to enable the Editor to make* The Hospital *of the utmost possible use and value to the profession. Specialists willing to review books on their special subjects are invited to communicate this fact at once. All letters should be addressed to* The Editor, The Lodge, Porchester Square, London, W.]

### MEDICINE.

### ALBUMINURIA.

Dr. Haig, physician to the Royal Hospital for Children and Women, contributes a lengthy article on "The Connecting Link between High-Tension Pulse and Albuminuria" (*British Medical Journal*, January 11th). Dr. Haig believes that the pre-albuminuric stage of Bright's disease is due to excess of uric acid in the blood. The high pulse tension of uric acid in the blood may be the absolute cause of renal disease. High arterial tension has an effect on the kidney circulation, and possibly also on its nutrition. This is intensified by deficient skin activity. Dr. Saundby gives cases of albuminuria lasting for years without kidney mischief becoming obvious. And Prof. Semmola gives the following sequence of causation. Deficient activity of skin function, leading to hereto-albuminæmia; that is, excess of diffusable albuminoids in the blood, these having failed to reach the condition of elaboration necessary for assimilation and the nutrition of tissues; this causing albuminuria, which, if continued, sets up changes in the structure of the kidney. Therefore, if these views are correct, Bright's disease is not the cause of albuminous urine, but caused by albuminous urine.

